# Accuracy of the Personal Economic Distress Index Among Arabic Unemployed

**DOI:** 10.3389/ijph.2023.1605851

**Published:** 2023-09-04

**Authors:** Khouloud Razki, Yosra Zgueb, Amina Aissa, Emna Sofia Ouali, Anis Wahabi, Uta Ouali

**Affiliations:** ^1^ Psychiatry Department (A) Razi Hospital, Manouba, Tunisia; ^2^ Faculty of Medicine, University Tunis El Manar, Tunis, Tunisia; ^3^ Psychiatry Department, Humanitas University, Rozzano, Italy; ^4^ Institut des Hautes Etudes Commerciales de Carthage, Tunis, Tunisia

**Keywords:** unemployment, psychometric properties, Arab, economic decline, COVID-19 pandemic

## Abstract

**Objectives:** Studies about The Index of Personal Economic Distress (IPED) in Arab countries since the onset of the COVID-19 pandemic have been alarming. This study aimed to explore the reliability, factor structure, and criterion validity of the Arabic IPED in a sample of unemployed Tunisians during the past 6 months.

**Methods:** The Arabic version of the IPED and the validated Hospital Anxiety and Depression Scale (HADS) were administered to a total of 2011 unemployed people originating from all Tunisian regions. Principal component analysis (PCA) with confirmatory factor analysis (CFA) was used to establish the spontaneous distribution of the 8 items and possible factors of the IPED. Receiver operating characteristic (ROC) analysis was conducted to assess the ability of the IPED to distinguish between unemployed individuals with no economic distress and those in distress. The correlation between the IPED and the HADS were explored.

**Results:** The reliability of the Arabic version of the IPED was adequate. The PCA suggested two main components of the Arabic version of the IPED: the first component including five items focused on financial responsibilities towards state structures and the second dimension containing three items focused on well-being. A statistically significant association (*p* = 0.01; r = 0.05) was found between the well-being dimension of the IPED and the Anxiety subscale of HADS.

**Conclusion:** This validated version of the IPED is an important tool to study the impact of economic crises on the mental health of unemployed people, as demonstrated in the link found in this study between economic distress and anxiety.

## Introduction

Since December 2019, the world has been facing an unprecedented health pandemic that started in China [1] and spread globally. This pandemic, with all the restrictive measures it implies, has not only caused a global humanitarian crisis, but also a severe global economic crisis, by creating an imbalance between supply and demand [2].

In this light, some economists have compared the socio-economic repercussions induced by the Coronavirus to those caused by the two world wars combined [2, 3]; the weakening of the employability of the workforce, the increase of public expenses, the drying up of tax revenues, and a significant increase of poverty and unemployment rates. In fact, the International Labor Organization reported that 20 million Americans became unemployed within weeks of the onset of the pandemic [4], which was considered a historical record. Also, according to reports from the Organization for Economic Cooperation and Development, a decline in global growth was estimated at 2.4% in 2020 as opposed to 2.9% predicted last year, following COVID-19 [5, 6].

The Tunisian economy, which had already been in continuous decline for a decade and in an unstable geopolitical atmosphere, became increasingly ambiguous and alarming since the onset of the COVID-19 pandemic [7]: 200,000 Tunisians have lost their jobs since November 2020 [8]. This economic situation further widens the gap between socioeconomic classes, while making the unemployed a disadvantaged social class with significant economic distress and increased vulnerability to anxiety and depressive disorders [9].

To our knowledge, in North Africa and especially in Tunisia, there is no objective measurement tool that allows researchers to assess the intensity of personal economic distress among the unemployed. Therefore, the evaluation is done in a purely subjective way. However, demands for scientific research in this field have prompted the use of objective assessment methods, and hence the need to develop a psychometrically validated scale is becoming increasingly important. In epidemiology, these instruments have the role of assessing the severity of the impact of the economic crisis on the unemployed. In clinical research, they facilitate the collection of socio-demographic data, the measurement of the severity of personal distress and the identification of prognostic elements of mental disorders.

Indeed, the Index of Personal Economic Distress (IPED) is an instrument widely used in studies done in Greece during the economic crisis [10], with well-documented effectiveness and relevance in the evaluation of the intensity of the economic distress in a targeted population, the unemployed and its correlations with mental disorders.

It is important to note that the process of cross-cultural validation of the IPED for Tunisia would take into consideration the psychosocial and cultural specificities of the study sample, the characteristic temperamental traits, as well as the unstable geopolitical situation.

Therefore, the goal of our study is to validate the Index of Personal Economic Distress (IPED) [10] on a sample of unemployed people in Tunisia.

## Methods

### Parameters and Participants

Our study took place over a period of 3 months, from July to September 2021, during the 3rd wave of the COVID-19 pandemic, where most of Tunisia was in lockdown. We used an online survey to select a group of unemployed citizens through Facebook using the snowball sampling technique.

First, we chose 10 suitable participants to fill out the questionnaire with necessary information. Then, each one of them eventually sent the same questionnaire to 10 other unemployed persons. This process allowed us to obtain a fair representation of unemployed citizens throughout the country (all 24 regions).

Our inclusion criteria were; (A) 18 as a minimum age, (B)Tunisian nationality, (C) Unemployed, which, according to the definition of the National Institute of Statistics (INS) and the International Labor office (BIT), is a person without a job during a given week; available to start a job within the next 2 weeks; actively having sought employment at some time during the last 4 weeks or having already found a job that starts within the next 3 months.

Questionnaires that contained duplicate answers, or were not fully filled out (less than 20%), were discarded and not taken into consideration.

The questionnaire guaranteed that all the responses to our survey were completely anonymous and offered participants the option to disengage from participating at any time. In addition, all participants were asked to inform us if they felt distressed at any point during or after filling out the questionnaire.

### Data Collection

We collected sociodemographic data anonymously and confidentially via online surveys (age, gender, marital status, geographic area, education level, employment status before job loss, living arrangement, housing situation and household monthly income before and during unemployment), personal medical history (history of follow-up for mental illness and history of suicidality), information on current financial status (duration of unemployment, family income, monthly benefits and social security coverage).

### Measures: Psychometric Tools

#### The Arabic Version of the Index of Personal Economic Distress (IPED)

IPED is a questionnaire that contains eight questions aiming to measure the degree of personal economic distress of unemployed people and describing their difficulty in fulfilling their daily household financial demands during the last 6 months.

The IPED scale contains 8 items: item 1 “Unable to pay the bills regularly (electricity, telephone, etc.),” item 2 “Difficulty paying bank loan installments,” item 3 “Incapable of paying the minimum credit card payment,” item 4 “Delays in paying rent,” item 5 “Cannot afford leisure activities,” item 6 “Cannot afford new clothing,” item 7 “Paying the minimum for food, beverages, etc.” and item 8 “Misses paying installment for the purchase of a car or other significant assets.”

All responses were made on a three-point-scale, reflecting frequency dimension: never (1), sometimes (2) and often (3).

The interpretation of the scale is done by adding the 8 items’ scores; the higher the score, the more intense the economic personal distress; ranging from 8 “no economic problem” to 24 “severe economic problem.”

To increase our tool’s sensitivity, to obtain more credible results, and to meet the user’s expectations, we added an additional answer “does not match (correspond),” not present in the original version, with a rating of 0 on the scale.

This revision allowed us to take into consideration the socio-economic context of Tunisia (lower middle-income country), as well as the users’ opinion, who openly reported their frustration by answering a few items that do not present a real difficulty in their daily lives (item 1, item 2, item 3, item 4 and item 8).

From a psychometric perspective, this modification was acceptable, as the additional response aimed to increase the scale’s sensitivity by giving the items a more continuous character.

The IPED’s translation took place over three stages according to the back-translation method proposed by Werner and Campbell and recommended by Brislin [11]. The translation’s adequacy and the adaptation of the items to the socio-cultural context of the final version of the scale were examined by the authors of the study and the committee of experts (experts in Arabic literature and experts in English literature).

#### Hospital Anxiety and Depression Scale (HADS)

The presence of depressive and anxious symptomatology was evaluated using the HADS designed by Zigmond A. S. and Snaith R. P. in 1983 [12]. We used the Arabic version, which was validated by A. Terkawi et al. in 2017 [13].

### Statistical Analysis

The sample was described using mean, standard deviation, median and percentage. A Spearman correlation was applied in order to determine the presence or absence of inter-item correlations, as well as to explore the test-retest’s reliability and consistency.

For the assessment of the questionnaire’s internal consistency, Cronbach’s Alpha was used. There is a consensus that scores above 0.7 indicate good internal consistency [14].

A confirmatory factor analysis (CFA) was carried out in order to examine the different dimensions of the scale. The IPED’s external validity was tested in relation to the economic status of the participant using the analysis of the receiver operating characteristic curve (ROC curve).

The participant’s economic status was assessed by the question “Do you feel you have been in economic distress for the last 6 months?”, and the answer was dichotomous (Yes, I am in economic distress/No, I am not in economic distress).

The data was computed and analyzed using the Statistical Package for the Social Sciences (SPSS) Version 26. In all tests, the statistical significance threshold (p) was set at 0, 05.

### Ethical Considerations

Study participants were provided with the numbers of all social services and as well as of therapists to contact if needed. Our study was approved by the Ethics Committee of Razi Hospital, Manouba (RPA 5/2022), authorization signed on August 10, 2022). It was conducted in accordance with the guidelines of the 1995 Helsinki Declaration and its revisions (General Assembly of the World Medical Association 2014).

## Results

### Socio-Demographic Data

We received a total of 2029 questionnaires for our study. Subsequently, 18 questionnaires were deemed invalid according to the exclusion criteria (more than 20% of the questionnaire left unanswered). Answering the various questions is mandatory, as per the regulations of the E-questionnaire platform; however, we added a “prefer not to say” option to each question to respect the privacy and right to non-response of the participants. Thus, the final sample size of our study was 2011 participants.

We did not observe any significant differences between the rejected responses and the included ones. The incomplete filling of the questionnaire could be explained by disinterest, lack of time, or the participants’ refusal to disclose personal data.

The percentage of rejected responses is 0.88% (N = 18) of the total responses, which did not significantly affect our results and conclusions.

The total sample consisted of 2011 participants, of which 73.8% (*n* = 1,484) were female with a sex ratio of 0.07. The average age of the study’s population was 27, 54 ± 5, 47 years, of which the most represented age group was 20–29 years (68.3%).

The majority of study participants had a university education (*n* = 1,744, 86.7%).

Regarding marital status, 1,574 participants (78.3%) were single, 397 participants (19.7%) were married, and 36 participants (15%) were divorced.

Also, 12% of the population reported that they had consulted a psychiatrist or psychologist once in their life, however only 6.7% are under medical treatment currently.

During the study, most of the participants had recently become unemployed (Table 1).

**TABLE 1:** Duration of unemployment in the study population (Tunisia, 2021).

	Percentage	Numbers
Less than a week	68.1%	1,369
Between 1 week and 1 month	6.1%	122
Between 1 month and 3 months	10.9%	220
More than 3 months	14.9%	300

The mean of the IPED for all study participants was 13.35 with a minimum of 0 and a maximum of 24, standard deviation was 6.86 and variance was 47.12. More than half of the study population (76.9%) had an overall IPED score higher than 8.

### Reliability

The Cronbach’s Alpha for the IPED scale is 0.89.

The correlations for the 8 items of the scale are reported in the third table, the Pearson coefficient “r” for the items 1,2,3,4 and 8 varies from 0.527 to 0.864, and for the items 5,6 and 7 varies from 0.537 to 0.697 as is shown in Table 2.

**TABLE 2:** Inter-item and inter-item-total correlation matrix of the IPED (Tunisia, 2021).

	Item1	Item2	Item 3	Item 4	Item 5	Item 6	Item7	Item 8	Totale-item
Item 1	1								
Item 2	0.683^a^	1							
Item 3	0.606^a^	0.864^a^	1						
Item 4	0.676^a^	0.753^a^	0.749^a^	1					
Item 5	0.359^a^	0.316^a^	0.333^a^	0.311^a^	1				
Item 6	0.410^a^	0.351^a^	0.375^a^	0.373^a^	0.697^a^	1			
Item7	0.466^a^	0.407^a^	0.440^a^	0.448^a^	0.537^a^	0.645^a^	1		
Item 8	0.527^a^	0.675^a^	0.681^a^	0.614^a^	0.396^a^	0.380^a^	0.379^a^	1	
Totale- Item	0.781^a^	0.851^a^	0.857^a^	0.837^a^	0.606^a^	0.654^a^	0.681^a^	0.783^a^	1

^a^ The correlation is significant at the 0.01 level (two-tailed).

### Test-Retest Stability

From the 2011 participants, a subset of 90 accepted a reassessment after 1 month.

The test-retest correlation was calculated for each item; item 1 (0.625), item 2 (0.450), item 3 (0.611), item 4 (0.413), item 5 (0.752), item 6 (0.622), item 7 (0.485) and item 8 (0.504), which indicates adequate reliability of IPED among the unemployed over time.

### Confirmatory Factor Analysis

The measure of KMO was 0.87, and Bartlett’s test of sphericity showed a significance *p* < 10-3.

**FIGURE 1:**
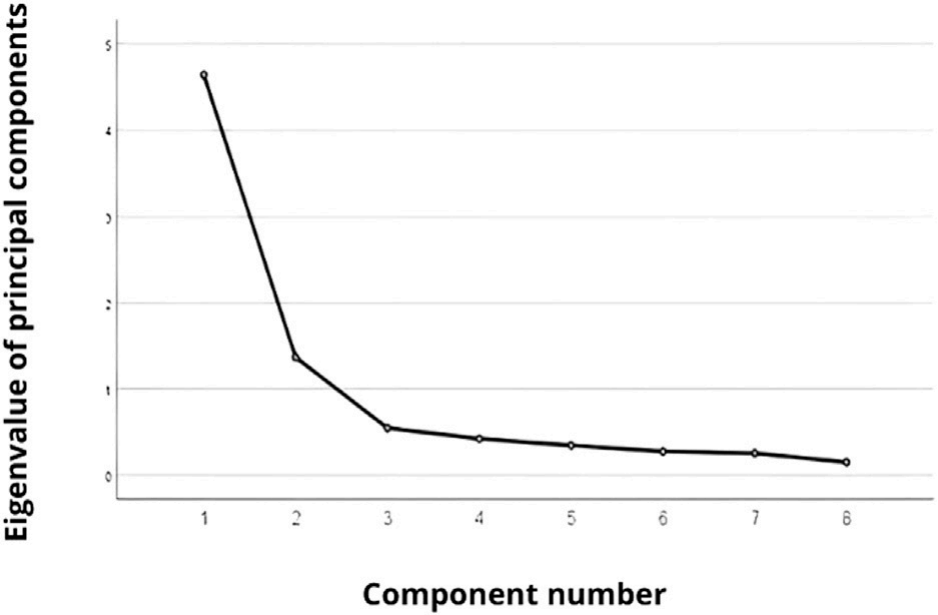
Principal component analysis (Tunisia, 2021).

A factor analysis of the 8 IPED items identified two factors with an eigenvalue that could correspond to two dimensions of the scale, explaining the 75.10% of the total variance (Figure 1).

**FIGURE 2:**
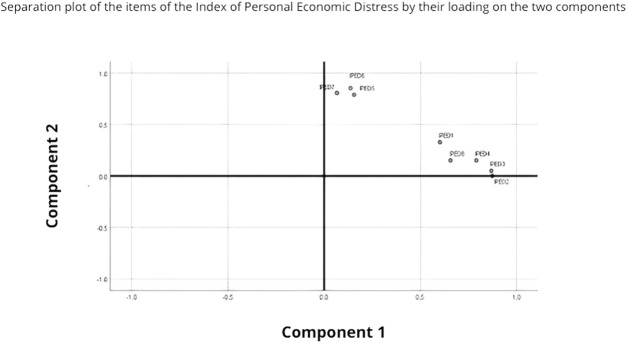
Tracing of the components in space after rotation (Tunisia, 2021).

The first factor clearly explains 58.01% of the total variance and the items are: 1, 2, 3, 4 and 8. The second factor explains 17.09% of the total variance and the items are: 5, 6 and 7 (Figure 2).

The inter-dimension correlations between the two components of the Arabic IPED scale are reported in Table 3.

**TABLE 3:** Correlation between IPED subscales using Spearman’s non-parametric test (Tunisia, 2021).

	Dimension 1: Item 1,2,3,4 et 8	Dimension 2: Item 5,6 et 7	IPED
Dimension 1: item 1,2,3,4 et 8	1		
Dimension 2: item 5,6 et 7	0.513^a^	1	
IPED	0.953^a^	0.748^a^	1

^a^ The correlation is significant at the 0.01 level (two-tailed).

### External Construct Validity

**FIGURE 3:**
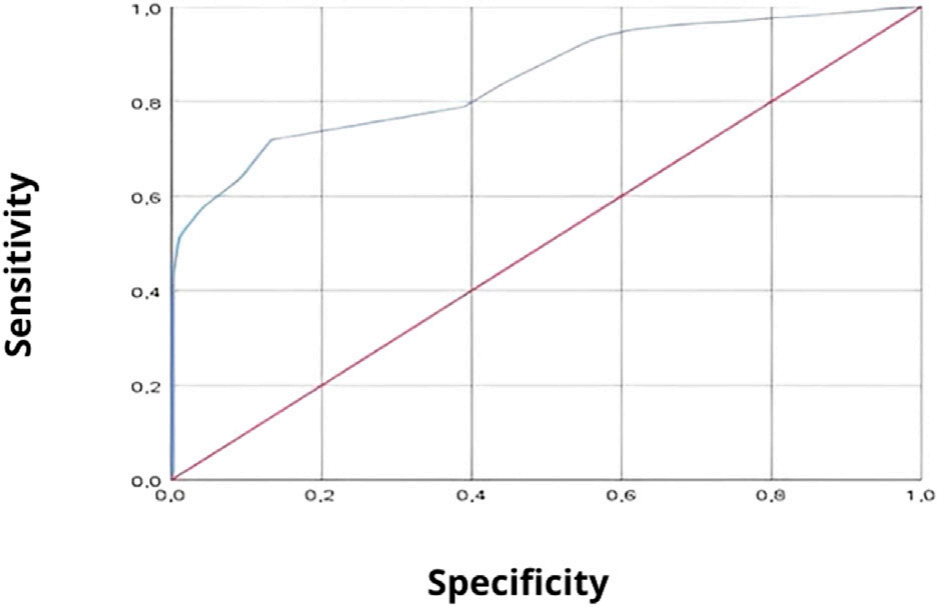
The Receiver operating characteristic curve of the Index of Personal Economic Distress scale (Tunisia, 2021).

The ROC curve provided the best cutoff point at 8.5 in order to produce the best sensitivity (71.9%; valid economic distress cases) and specificity (86.7%; valid economic well-being cases) values (Figure 3).

The area under the ROC curve (AUC) was estimated to be 0.848 with a confidence interval [0.830–0.866].

### The Relation Between IPED Subscales and Anxiety-Depressive Disorders

We examined the statistical associations between the different IPED dimensions and the HADS (Anxiety, Depression) subscales in Table 4.

**TABLE 4:** Statistical associations between IPED and HADS (Tunisia, 2021).

	HADS: Anxiety subscale	HADS: Depression subscale
Dimension 1: item 1,2,3,4 et 8	*p* = 0.713	*p* = 0.563
	R = 0.008	R = −0.013
Dimension 2: item 5,6 et 7	*p* = 0.015	*p* = 0.172
	R = 0.054	R = − 0.030
IPED	*p* = 0.05	*p* = 0.943
	R = 0.046	R = 0.002

It appears that the more participants had difficulties in meeting financial demands regarding leisure activities (item 5), shopping expenses (item 6) and groceries (item 7), the more anxious symptoms were present (χ2 = 5.890, p (Fisher test) = 0.016, OR = 1.24, CI 95% [1.04–1.49]).

## Discussion

The goal of our study was to validate the Arabic version of the IPED personal economic distress estimation questionnaire in a sample of Tunisian unemployed people.

The Arabic version of the IPED demonstrates good internal consistency, with a Cronbach’s alpha coefficient estimated at 0.89. Each item shows a statistically significant positive correlation with other items and the total score of the IPED. Therefore, all items were retained and included in the multifactorial analysis using principal component analysis. Test-retest reliability analysis confirms that our version of the IPED scale remains stable over time, with correlations ranging from 0.413 to 0.752. The reliability of the Arabic version of the IPED was deemed adequate. The ROC curve provided the optimal cutoff point of 8.5 to yield the best sensitivity (71.9%, true cases of economic distress) and specificity (86.7%, true cases of economic well-being) values. The area under the ROC curve (AUC) was estimated at 0.848 with a confidence interval of [0.830–0.866]. Principal component analysis suggested two main components of the Arabic version of the IPED:

• The first component consists of five items focusing on financial responsibilities towards state structures, such as bill payments, rent, and bank loans.
• The second dimension includes three items related to well-being, such as good food, leisure activities, and shopping.

Our results indicate that the Arabic version of the IPED has good internal consistency, Cronbach’s alpha was estimated at 0.89, very close to that of the original instrument (alpha = 0.81) [10].

Each item had a statistically significant positive correlation with the other items and with the total IPED score. Therefore, all items were kept and included them in the multifactorial study by principal component analysis [15].

In line with the original article [10], the test-retest reliability analysis confirms that our version of the IPED scale remains stable over time with correlations ranging from 0.413 to 0.752.

The results of the CFA of the Arabic version of the IPED identified two factors with an eigenvalue that could correspond to two dimensions of the scale explaining 75.10% of the total variance: Dimension 1 (item 1,2,3,4 and 8) focused on financial responsibilities towards state structures and Dimension 2 (items 5,6 and 7) focused on well-being.

68.3% More than two thirds of our study sample (68.3%) was between 20 and 29 years, whereas the majority (73.3%) of the participants in the study of the original Greek article [10] was above 35 years. This difference in age between the two study populations could explain the difference between interest and lifestyle. Indeed, young people tend to be more interested in shopping (item 6), leisure activities (item 5) and going out with friends to try new things, which is usually not the case for older people who become more financially responsible by having critical tasks such as: paying bills (item 1), paying rent (item 4), credit card fees (item 3) and car fees (item 8) or repaying bank loans (item 2).

In addition, the model of European and Maghrebian families is not the same. In Western society [16, 17], the family structure is usually more modern and liberated, giving young people more independence (housing, creating business) than in North African society. Also, in Tunisia, despite the modernization that the family model has undergone in recent decades, the relational dynamic between parents and their children tends to retain a conservative, traditional, paternalistic and even hyper-protective aspect which does not encourage young people to adopt an autonomous lifestyle (housing and financial independence) [18]. In this light, more than three quarters of the participants in our study were living in their parent’s home and were financially supported by them, which could explain the subdivision of the IPED into two dimensions.

We found a statistically positive correlation between the D2 dimension of the IPED and the HADS subscale (Anxiety) (*p* = 0.015 and r = 0.054). This expected result could be explained by several biopsychosocial factors. According to the study by Chebbi et al. [19], the hyperthymic temperament (9.95 ± 4.39) is the basis of the Mediterranean temperament of the Tunisian population, followed by the anxious temperament (9.03 ± 5.37), cyclothymic temperament (8.35 ± 4.72), depressive temperament (8.34 ± 3.15), and irritable temperament (4.82 ± 3.84). However, EG. Karam et al. [20] reported in their studies that social judgment disorder and financial problems contribute to dimming the brightness of this temperament and altering the mental health of the subjects by contributing to frequent anxiety comorbidity (panic attacks, separation anxiety). In this light, we explain the positive correlation between the subscale (anxiety) and the D2 dimension of the IPED, whose items were focused on sociability, extravagance (good clothing) and exploratory excitability (leisure activity). Secondly, we find several studies in the literature [21, 22] which have shown that the prevalence of anxiety disorders is greater in young people than in older people. This could explain our result since the majority of the participants (68.3%) were aged between 20 and 29 years old.

However, we did not find a statistical association between the IPED dimensions and the HADS subscale (depression). Indeed, as mentioned above, the traditional and paternalistic model of family in Tunisia [18] provides young people with solid emotional and financial support, decreasing the mental burden and worries of the unemployed. In other words, families continue to meet the primary needs of their children even in adulthood, which creates a situation of financial security for the unemployed. Also, during the last decade, Tunisian society has developed coping skills to face the economic situation while normalizing the status of the unemployed. In fact, the number of unemployed estimated for the first quarter of 2022 reached 653.2 thousand of the total working population with a rate of 16.2% according to the latest statistics [23], so unemployment has ceased to be a marginalized social phenomenon and is increasingly becoming a reality experienced by consecutive generations. The young unemployed, for their part, continue to work temporary jobs (freelance, call centers, delivery, waiters, etc.) in order to maintain a certain dynamic of socio-professional functioning and to reserve a social role that forges their integration into their social core. Thus, the idea of immigration to European countries has become the common and urgent occupation of young Tunisians, especially those who are unemployed. In fact, the immigration rate in 2020 was estimated at 7.6% of the total population, or 900,000 migrants [24], whose search for work represents the main reason for the desire to immigrate (51.7%), followed by the search for better standards and living conditions (26.7%). In the same way, the preparation of immigration files, learning languages and new skills fosters optimism and fights against depressive cognitions among the unemployed. They continue to project themselves into the future and have a positive view of the world outside their native country.

### Strengths and Limitations

To the authors’ knowledge, this is the first cross-cultural validation of the IPED scale study in North Africa to focus on the mental health of the unemployed and its links with socioeconomic difficulties, especially during a period of sustained financial recession (the economic crisis following the COVID-19 pandemic). Among the methodological strengths of our study is the fact that anxiety-depressive symptomatology was assessed using a clinical tool (HADS) rather than a screening test.

Nevertheless, the study has some limitations that deserve consideration.

First of all, we cannot exclude the possibility of a recruitment bias, since we conducted our study with unemployed people only, and therefore do not have a control sample. In addition, as the mode of sampling and data collection was a self-questionnaire distributed online, it is likely that the unemployed with great economic difficulties have neither the means nor the access to the Internet and were therefore excluded from the study. This limitation could have been circumvented if the survey had been done among households, but this was not possible as recruitment was done at the time of the 4th wave of COVID-19 in Tunisia, while respecting the means of restrictions imposed by the Ministry of Health and by WHO.

Similarly, a non-response or false response bias may have occurred. In fact, we cannot exclude the possibility of false answers concerning the economic status of the participant and the extent of the daily financial difficulty (e.g., due to a feeling of shame). Thus, the lack of direct contact between the participants and the authors of the study may have biased the answers, since the understanding of certain questions depends partly on the perception of each participant, and this may create nuances while answering.

### Conclusion

The Arabic IPED has similar psychometric properties to the original instrument: good internal consistency, excellent inter-rater reliability for the overall score and good temporal stability. Our statistical analysis showed a two-factor structure, which allowed us to group the IPED items into two dimensions (financial responsibilities and well-being). We concluded that there was a clear difference in the perception of economic distress between the two dimensions by the unemployed.

In fact, we found from the second dimension of the IPED in its validated version in Arabic that a good diet, shopping, and leisure are the cornerstone of well-being of the Tunisian unemployed, even in the presence of other financial difficulties.

Thus, the socio-cultural adaptation of the IPED has created a version that is faithful to the mosaic of the bio socio-cultural characteristics of the Maghrebi population.

As a result, our study contributes to the development of a reliable tool for measuring personal economic distress in order to facilitate the conduct of future studies, especially considering the impact of the political and economic crisis on the mental health of individuals.

## References

[1] Khan WH Hashmi Z Goel A Ahmad R Gupta K Khan N COVID-19 Pandemic and Vaccines Update on Challenges and Resolutions. Front Cell Infect Microbiol (2021) 11:690621. 10.3389/fcimb.2021.690621 34568087 PMC8461057

[2] Nicola M Alsafi Z Sohrabi C Kerwan A Al-Jabir A Losifidis C. The Socio-Economic Implications of the Coronavirus Pandemic (COVID-19): A Review. Int J Surg (2020) 78:185–93. 10.1016/j.ijsu.2020.04.018 32305533 PMC7162753

[3] Roy S Das D. Economic Impact of COVID-19 Pandemic (2020). Available from: https://www.researchgate.net/publication/343222400_ECONOMIC_IMPACT_OF_COVID-19_PANDEMIC?enrichId=rgreq-f9fe092c9d45e749d2191a7877009e96-XXX&enrichSource=Y292ZXJQYWdlOzM0MzIyMjQwMDtBUzo5MTc3MDYxMTUxMjExNTJAMTU5NTgwOTU5NjMzOA%3D%3D&el=1_x_2&_esc=publicationCoverPdf (Accessed December 18, 2022).

[4] Kozicki B Gornikiewicz M. Unemployment Rate in Poland and USA During COVID-19 Pandemic: A Case Study. Eur Res Stud J (2020) XXIII:187–200. 10.35808/ersj/1861

[5] World Bank. Global Economic Prospects. Washington, DC: The World Bank (2020). p. 234.

[6] OECD. Unemployment Rates, OECD - Updated: April 2021 (2021). Available from: https://www.oecd.org/sdd/labour-stats/unemployment-rates-oecd-update-april-2021.htm (Accessed October 10, 2022).

[7] OECD. Tunisia Economic Snapshot - OECD (2022). Available from: https://www.oecd.org/economy/tunisia-economic-snapshot/(Accessed October 10, 2022).

[8] Statista. Tunisia - Unemployment Rate 1999-2021 (2023). Available from: https://www.statista.com/statistics/524516/unemployment-rate-in-tunisia/ (Accessed October 10, 2022).

[9] Boyd JH Weissman MM. Epidemiology of Affective Disorders: A Reexamination and Future Directions. Arch Gen Psychiatry (1981) 39(9):1039–46. 10.1001/archpsyc.1981.01780340091011 7025783

[10] Medianos M Economou M Alexiou T Stefanis C. Depression and Economic Hardship Across Greece in 2008 and 2009: Two Cross-Sectional Surveys Nationwide. Soc Psychiatry Psychiatr Epidemiol (2011) 46:943–52. 10.1007/s00127-010-0265-4 20614103

[11] Brislin RW. Back-Translation for Cross-Cultural Research. J Cross-Cultural Psychol (1970) 1:185–216. 10.1177/135910457000100301

[12] Zigmond AS Snaith RP. The Hospital Anxiety and Depression Scale. Acta Psychiatr Scand (1983) 67 (6):361–70. 10.1111/j.1600-0447.1983.tb09716.x 6880820

[13] Terkawi AS Tsang S AlKahtani GJ Al-Mousa SH Al Musaed S AlZoraigi US Development and Validation of Arabic Version of the Hospital Anxiety and Depression Scale. Saudi J Anaesth Mai (2017) 11(1):S11–S18. 10.4103/sja.SJA_43_17 PMC5463562 28616000

[14] Taber KS. The Use of Cronbach’s Alpha When Developing and Reporting Research Instruments in Science Education. Res Sci Edu (2018) 48:1273–96. 10.1007/s11165-016-9602-2

[15] Streiner DL Norman GR Cairney J. Health Measurement Scales: A Practical Guide to Their Development and Use. United Kingdom: Oxford University Press (2015). p. 415.

[16] Gianesini G. European Families: Structures, Policies and Social Trends. In: Shehan CL, editor. The Encyclopedia of Family Studies. United States: Wiley (2014).

[17] Kraus B Ondrejkovič P Krzysztof Świątkiewicz W Vilka L Rieke U Trapenciere I Characteristics of Family Lives in Central Europe. In: Kraus B Stašová L Junová I, editors. Contemporary Family Lifestyles in Central and Western Europe: Selected Cases. Cham, Berlin, Germany: Springer International Publishing (2020). p. 21–47. (SpringerBriefs in Sociology) Disponible sur. 10.1007/978-3-030-48299-2_2

[18] Mokounkolo R Taillandier-Schmitt A. Sociodemographic and Psychological Variables Related to Sociocultural, Acculturative Orientation of North African Immigrants in France. South Afr J Psychol (2008) 38:408. 10.1177/008124630803800408

[19] Chebbi W Khalfaoui S Gassab L Mechri A. Assessment of Affective Temperaments in a Tunisian Non-Clinical Population. Tunis Med (2019) 97(11):1277–83. 32173831

[20] Karam EG Salamoun MM Yeretzian JS Mneimneh ZN Karam AN Fayyad J The Role of Anxious and Hyperthymic Temperaments in Mental Disorders: A National Epidemiologic Study. World Psychiatry (2010) 9(2):103–10. 10.1002/j.2051-5545.2010.tb00287.x 20671899 PMC2911090

[21] Bandelow B Michaelis S. Epidemiology of Anxiety Disorders in the 21st Century. Dialogues Clin Neurosci (2015) 17(3):327–35. 10.31887/DCNS.2015.17.3/bbandelow 26487813 PMC4610617

[22] Jacobi F Höfler M Strehle J Mack S Gerschler A Scholl L Mental Disorders in the General Population: Study on the Health of Adults in Germany and the Additional Module Mental Health (DEGS1-MH). Nervenarzt (2014) 85(1):77–87. 10.1007/s00115-013-3961-y 24441882

[23] Statista. Tunisia: Unemployment Rate by Quarter 2019-2022 (2023). Available from: https://www.statista.com/statistics/1237604/unemployment-in-tunisia-by-quarter/ (Accessed October 10, 2022).

[24] ETF_Skills. Skills and Migration Country Fiche Tunisia (2021). Available from: https://www.etf.europa.eu/sites/default/files/2021-11/etf_skills_and_migration_country_fiche_tunisia_2021_en_1.pdf (Accessed October 10, 2022).

